# Suicidality as a potential risk and therapeutic target during widespread GLP-1 receptor agonists use: implications of inflammation

**DOI:** 10.3389/fnsyn.2026.1869086

**Published:** 2026-06-04

**Authors:** Milica M. Borovcanin, Ivica Petrovic

**Affiliations:** 1 Department of Psychiatry, Faculty of Medical Sciences, University of Kragujevac, Kragujevac, Serbia; 2 Psychiatric Clinic, University Clinical Centre Kragujevac, Kragujevac, Serbia; 3 Department of Pathophysiology, Faculty of Medical Sciences, University of Kragujevac, Kragujevac, Serbia

**Keywords:** dopamine, GABA, ghrelin, GLP-1 RA, inflammation, leptin, suicidality

## 1 Introduction

The use of Glucagon-Like Peptide-1 Receptor Agonists (GLP-1 RAs) has revolutionized the treatment of Type 2 Diabetes Mellitus (T2DM). However, there is growing pressure from patients and healthcare professionals in various fields to use these drugs beyond approved indications to achieve benefits across multiple organ systems—cardiovascular, musculoskeletal—and to obtain anti-inflammatory effects. Their indications have since expanded to include obesity and metabolic syndrome X, fundamentally changing the medical paradigm. This topic falls within the field of translational medicine, as it seeks to integrate basic research into clinical practice. Yet, it also casts a shadow. Balancing desired benefits and potential risks, including those affecting mental health, remains essential. No substance is so selective that it produces only the desired effects without disturbing another balance in the organism. Research to date and its findings indicate the need for a comprehensive and complementary approach to the use of these drugs, especially regarding a potential link between their mechanisms of action, observed effects, and suicidality risk.

## 2 Comorbidity of diabetes and mental disorders

In our previous work, we discussed whether T2DM underlies mental disorders, exists independently or in parallel with them, or may arise as a consequence of mental disorders (Borovcanin et al., 2023). For psychiatrists, T2DM is a frequent concern when, for example, introducing atypical antipsychotics to patients. These medications help in the prevention of various extrapyramidal symptoms by sparing dopamine but present new challenges, such as metabolic syndrome, hyperlipidemia, hyperglycemia, and T2DM (Bhardwaj et al., 2025). In consultative work within endocrinology departments, attending physicians most often observe psychotic symptoms or cognitive changes in patients with poorly controlled glycemia, even leading to delirium. These changes may be transient, due to hypo or hyperglycemia, or persistent, and require consultation with psychologists and psychiatrists. Interestingly, the association between anxiety and T2DM was noted with the Gamma-AminoButyric Acid (GABA) analog pregabalin prescribing. Initially, it was approved for the treatment of neuropathic pain associated with diabetic peripheral neuropathy and postherpetic neuralgia, and later tested in a clinical trial for generalized anxiety disorder (Tassone et al., 2007). This highlighted similar neurotransmission substrates in the development of these disorders. A German study showed that the prevalence of affective disorders in patients with T2DM is twice as high as in those without T2DM (Hermanns et al., 2005). Further, and maybe the most important, depression, with its risk of suicidality, must also be considered, especially in patients with T2DM as an endpoint of the metabolic disorders' spectrum.

## 3 Inflammation and GLP-1 receptors in metabolic disorders and depression

Inflammation is increasingly recognized as a therapeutic target in various neuropsychiatric disorders, linked to immunometabolic changes. T2DM must be considered as consequent to obesity and is also associated with acute and chronic stress and systemic proinflammatory state. These mechanisms further activate the hypothalamic-pituitary-adrenal axis, hepatic glycogenolysis, hyperglycemia, hyperlipidemia, and insulin resistance, contributing to core depressive features of anhedonia, emotional dysregulation, and cognitive slowing (Sarwar et al., 2022). Thus, it is clear that the focus is not solely on glycemic regulation, but rather on immunometabolism, leading to possible depression onset. Animal models have also identified interaction pathways between the brain and adipose tissue, as well as between the brain and the microbiota, indicating that peripheral events strongly influence central nervous system activity and behavior (Kim et al., 2015; Kanji et al., 2018; Li et al., 2022).

The complex etiopathogenesis of T2DM has been explored in detail and includes insulin resistance (in the liver, muscles, and adipose tissue), impaired insulin secretion, β-cell dysfunction, immune dysregulation and meta-inflammation, hyperglucagonemia, disrupted gut microbiota, increased gastric glucose absorption, renal adaptation with enhanced glucose reabsorption and gluconeogenesis, decreased dopamine, and heightened sympathetic tone in the brain (d'Aiello et al., 2023). In this context, the basis for the action of the new generation of drugs is the reduced secretion and/or less effectiveness of the gastrointestinal hormone Glucagon-Like Peptide-1 (GLP-1). This hormone regulates pancreatic islet function, enhances insulin production, reduces glucagon production, and modulates satiety and gastric motility. Its secretion is disturbed in patients with T2DM (McLean et al., 2021). Activation of GLP-1 Receptors (GLP-1 R) produces hypophagic effects in the ventral hippocampus, and GLP-1 RAs suppress appetite *via* cells of the circumventricular organs located around the third and fourth brain ventricles, transmitting signals to deeper brain structures. Since these receptors are present in various organ systems and in the brain, their roles in satiety and appetite regulation, as well as neuroinflammation and neuroplasticity, must be considered. These compounds reduce central neuroinflammation by lowering interleukin (IL)-1β, IL-6, and tumor necrosis factor-alpha (TNF-α), and limiting oxidative stress (Carmellini et al., 2025).

On the other side, current trends in precision psychiatry aim to classify subpopulations of patients with inflammatory depression, characterized by elevated markers of inflammation, increased glucose and lipid levels, neurovegetative symptoms, anxiety or irritability, and suicidal ideation (Miller, 2025). This suggests that therapeutic strategies for this specific depression subtype should target inflammation, immunometabolism, and neurotransmitter metabolism. In this context, the use of GLP-1 RAs in this subpopulation is considered justified (Wessa et al., 2026). Dual strategies that address both metabolic disorders and depression simultaneously appear reasonable.

## 4 GLP-1 receptor agonists and suicidality

Semaglutide, dulaglutide, liraglutide, exenatide, and tirzepatide are administered daily or weekly, either by injection or in tablet form, with limited approval for children over 10 or 12 years of age, or only adults, for T2DM or weight reduction, offering additional benefits for the heart and kidneys. The use of GLP-1 RAs is increasingly common, not only for T2DM but also for obesity in both youth and adults, though it is associated with numerous registered serious adverse effects.

Conversely, suicidal thoughts, intentions, self-harm, attempts, and completed suicides are the focus of consultative psychiatry in patients prescribed GLP-1 RAs. Regarding suicidality risk, recommendations for GLP-1 RAs use were issued by the U. S. Food and Drug Administration in 2024 as a warning (U. S. Food and Drug Administration, 2024) and in 2026 as a risk-removal label (U. S. Food and Drug Administration, 2026). The European Medicines Agency issued a 2024 notice stating no evidence of association (European Medicines Agency, 2024). The link between GLP-1 RAs use and suicidality is reflected in a higher risk among obese patients for major depression, anxiety, and suicidal behavior (Kornelius et al., 2024). World Health Organization pharmacovigilance data show variable associations with depression and suicidality depending on the agent used (Ruggiero et al., 2024). Meta-analyses did not find differences compared with other drugs for T2DM, though a higher risk was noted with liraglutide (McIntyre et al., 2025). Among the GLP-1 RAs, there are variations, such as the predominance of semaglutide over liraglutide in relation to the less frequent occurrence of suicidality (Alansari et al., 2025).

Retrospective cohort study of obese or T2DM patients reported cases showing no link—or even improvement—in suicidality, with reduced risk of sudden or recurrent suicidal thoughts (Wang et al., 2024). A large cohort study of T2DM patients using active comparators found no difference (Shapiro et al., 2025). Genetic proxy activation of GLP-1 RAs showed reduced risk for anxiety, depression, or emotional lability (Zheng et al., 2025). Data mining has not shown signals of association with mood disorders. A French nationwide case-time-control study of completed suicides and suicide attempts found no specific risk related to prior psychiatric history or obesity during GLP-1 RA use (Bezin et al., 2024).

## 5 State of the art and future directions

No clear causal mechanism linking GLP-1 RA use to suicidality has been established. Most discussions of the previously presented robust data analyses have focused on the hypothalamic-pituitary-adrenal axis and neurotrophic factors. All that imposes to inflammation as a missing link and possible explanation for beneficial and also unwanted effects of GLP-1 RA use. A simplified explanation is that inflammation could be ameliorated with these drugs. But we do not completely know what happens when the previous pathological and longstanding balance is abruptly disturbed in different cascades of the chronic stress and reward system, involving leptin and ghrelin, GABA and dopamine, altogether with smoldering inflammation.

The administration of GLP-1 enhances the feeling of satiety. It indirectly prolongs fasting periods, reduces meal frequency, and decreases meal-initiation pleasure via dopamine pathways. GLP-1 also reduces meta-inflammation and has an inhibitory effect on leptin resistance. This increase in the feeling of satiety, provided by leptin, leads to leptin blocking ghrelin and reducing the indirect feeling of pleasure during food intake. Ghrelin acts on reward-related pathways by stimulating dopamine neurons in the ventral tegmental area and promoting dopamine turnover in the nucleus accumbens, a region of the ventral striatum (Jerlhag et al., 2007). Further, its effects in the mesolimbic dopamine system contribute to its orexigenic action (Abizaid et al., 2006) and suppresses orexigenic neurons through GABA-dependent signaling (Huang et al., 2019). Reduced orexigenic activity is associated with improved sleep (Ribeiro et al., 2025), which is essential for alleviating depression and decreasing suicidality. Insomnia may contribute to suicidality and depression by increasing inflammation in the body (Xerfan et al., 2026), implicating that the anti-inflammatory properties of GLP-1 RAs could also be beneficial.

Preclinical models indicate that the GLP-1 R system has evolved, at least in part, to regulate feeding-related motivation (Gao et al., 2026). Gao and colleagues recently presented a fascinating interpretation of GLP-1 R signaling. When energy balance is positive, GLP-1 RA dampens activity in the brain's reward circuitry and reduces effort-based food seeking, a phenomenon also observed in other motivated behaviors. However, GLP-1 R signaling seems to influence affective behavior depending on energy levels. This could lead from exploration to conservation, shifting external exploration toward internal regulation as a short-term effect. Conversely, long-term treatment with GLP-1 RAs promotes anxiolytic and antidepressant effects rather than a fleeting interoceptive response.

Repeated dosing is probably crucial for positive effects in depression and suicidality. The beneficial effects of GLP-1 RAs are primarily rooted in endocrine physiology rather than willpower failure (Aronne et al., 2024). Guidelines indicate that these medications show their effects only after extended periods of consistent dosing, without complete discontinuation (Gasoyan et al., 2025). Following intensive therapy with tirzepatide, it is necessary to continue with therapy to promote long-term weight maintenance, especially in associated comorbid T2DM (Apovian et al., 2015). Prolonged dosing could be beneficial in those non-responders in an attempt to ameliorate depressive symptoms and suicidality.

Longitudinal observation is needed in different subpopulations of patients. New studies are being designed and conducted to evaluate potential suicidality or depressive symptoms associated with GLP-1 RAs prescriptions. In the 8-week Phase II trial of tirzepatide, depressive symptoms will be evaluated in patients with obesity and alcohol use disorder (Morley, 2025, http://ClinicalTrials.gov ID NCT07292519). In the Phase IV study, semaglutide will be evaluated for its effects on improving depressive symptoms over 26 weeks in patients with major depressive disorder who also have overweight or obesity (Vinberg, 2026, http://ClinicalTrials.gov ID NCT07136714). It will be particularly insightful to study the impact of GLP-1 RAs on affective symptoms during the maintenance phase or after treatment is discontinued.

**Figure 1:**
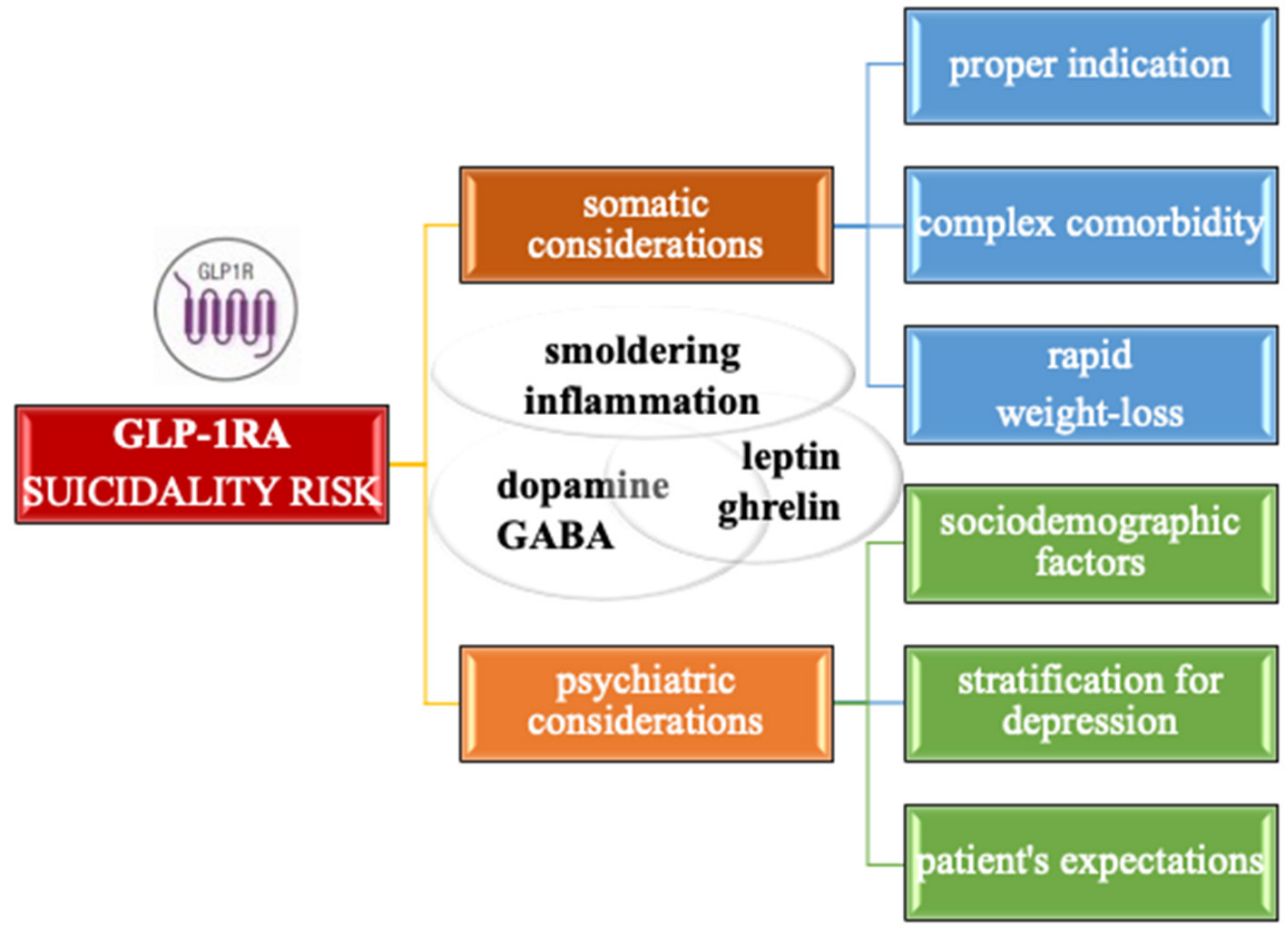
Proposed algorithm for prescribing GLP-1 RAs and exploring new therapeutic targets related to suicidality. When prescribing GLP-1 Receptor Agonists (GLP-1 RAs), both somatic and psychiatric factors should be considered. An adequate somatic diagnosis should be established, somatic comorbidities assessed, and the appropriate GLP-1 RA selected for each patient. The patient's somatic status and potential adverse reactions require careful monitoring throughout follow-up, particularly following rapid weight loss. Sociodemographic characteristics, prior mental health history, and patient expectations should be discussed and considered during ongoing monitoring of patients receiving GLP-1 RAs. Stratification for depression, either present at initiation or emerging as a consequence of treatment, may inform therapeutic decisions. Conversely, long-term treatment with GLP-1 RAs promotes anxiolytic and antidepressant effects rather than a fleeting interoceptive response. This Figure highlights the need for future research on the reward cascades involving leptin and ghrelin, dopamine and Gamma-AminoButyric Acid (GABA) regarding suicidality in GLP-1 RAs prescribing.

Long-term study results are currently limited, but the following must be considered for shaping the clinical algorithm in GLP-1 RA prescribing: proper indication, the characteristics of the prescribed drug, the patient's psychiatric and somatic history, sociodemographic factors, patient expectations, and especially monitoring of response to rapid weight loss (Figure 1).

Basically, in drug prescribing, the triad must be followed. Firstly, the response profile is very much influenced by the properties of the specific GLP-1 RA drug. Secondly, the majority of the studies are exploring only GLP-1 RA prescribing, but the patient's primary somatic diagnosis must be considered crucial. The various responses on GLP-1 RAs in patients with T2DM or obesity or both, and even additional comorbidities, must be more thoroughly examined. And, as a third, mental disturbances or even a diagnosis of a mental disorder must be taken into account. Patients can usually be divided into two groups when starting GLP-1 RAs: those with depression and those without. It could be suggested to pay attention to all adverse events, but especially mood disorders and suicidality risk. Schizophrenia could also be a context for implementing these immunometabolic strategies (Ganeshalingam et al., 2025).

Previous clinical observations indicate that a rapid and substantial reduction in body mass may result in significant mental disturbances. GLP-1 RA suppresses appetite and food reward to elicit rapid weight loss. In bariatric surgery, an association of depression with peripheral inflammation was observed regardless of obesity (McLaughlin et al., 2024). Further, intensified inflammation at baseline predicted poorer weight loss 6 months after surgery, regardless of depression diagnosis.

The new challenge of GLP-1 RA use reinforces that only an integrative approach is justified in treating each individual patient. The endocrinologist must be informed and include the basic psychological explorations and referral to a psychiatrist if psychopathology is observed. This must be done at the start and longitudinally, through the treatment. All aspects, desired but also unwanted effects of these drugs must be presented to the patients, and active cooperation is necessary. Individual concerns and expectations must be discussed because unrealistic aims could lead to greater frustrations (Ṣtefǎnescunescu et al., 2025). The effects of these drugs beyond the desired outcomes should not be minimized—they are far more potent than the marketing concept of “GLP-1 care”.
